# Supplementary material for: Longitudinal trajectories of brain age in young individuals at familial risk of mood disorder from the Scottish Bipolar Family Study
Source: Wellcome Open Res. 2020 Nov 9;4:206. Originally published 2019 Dec 17. [Version 3] doi: 10.12688/wellcomeopenres.15617.3 (PMC7479500; doi:10.12688/wellcomeopenres.15617.3)
Supplement: Peer review history

# Peer review: Reviewer response for version 3

**DOI:** 10.21956/wellcomeopenres.18057.r41346

**Published:** 2020-11-11

**Authors:** Janice Fullerton

**Affiliations:** 1 Neuroscience Research Australia, Sydney, NSW, Australia

I thank the authors for addressing the concerns I raised in my peer review report, and including the additional supplementary graphs which clearly indicate no effect of MRI quality on brain-PAD.

Is the work clearly and accurately presented and does it cite the current literature?

Yes

If applicable, is the statistical analysis and its interpretation appropriate?

Partly

Are all the source data underlying the results available to ensure full reproducibility?

Partly

Is the study design appropriate and is the work technically sound?

Yes

Are the conclusions drawn adequately supported by the results?

Partly

Are sufficient details of methods and analysis provided to allow replication by others?

Yes

Reviewer Expertise:

Young people at increased genetic risk of bipolar disorder

I confirm that I have read this submission and believe that I have an appropriate level of expertise to confirm that it is of an acceptable scientific standard.

# Peer review: Reviewer response for version 3

**DOI:** 10.21956/wellcomeopenres.18057.r41345

**Published:** 2020-11-11

**Authors:** Philip B. Mitchell

**Affiliations:** 1 School of Psychiatry, University of New South Wales, Sydney, NSW, Australia

I approve this revision.

Is the work clearly and accurately presented and does it cite the current literature?

Yes

If applicable, is the statistical analysis and its interpretation appropriate?

Yes

Are all the source data underlying the results available to ensure full reproducibility?

No source data required

Is the study design appropriate and is the work technically sound?

Yes

Are the conclusions drawn adequately supported by the results?

Partly

Are sufficient details of methods and analysis provided to allow replication by others?

Yes

Reviewer Expertise:

Young people at increased genetic risk of bipolar disorder

I confirm that I have read this submission and believe that I have an appropriate level of expertise to confirm that it is of an acceptable scientific standard.

# Peer review: Reviewer response for version 2

**DOI:** 10.21956/wellcomeopenres.17743.r39781

**Published:** 2020-09-14

**Authors:** Philip B. Mitchell

**Affiliations:** 1 School of Psychiatry, University of New South Wales, Sydney, NSW, Australia

The authors have addressed my concerns. No further work required.

Is the work clearly and accurately presented and does it cite the current literature?

Yes

If applicable, is the statistical analysis and its interpretation appropriate?

Yes

Are all the source data underlying the results available to ensure full reproducibility?

No source data required

Is the study design appropriate and is the work technically sound?

Yes

Are the conclusions drawn adequately supported by the results?

Partly

Are sufficient details of methods and analysis provided to allow replication by others?

Yes

Reviewer Expertise:

Young people at increased genetic risk of bipolar disorder

I confirm that I have read this submission and believe that I have an appropriate level of expertise to confirm that it is of an acceptable scientific standard.

# Peer review: Reviewer response for version 2

**DOI:** 10.21956/wellcomeopenres.17743.r40196

**Published:** 2020-09-08

**Authors:** Janice Fullerton

**Affiliations:** 1 Neuroscience Research Australia, Sydney, NSW, Australia

This work explores an interesting and topical research area in a cohort of young individuals at risk of mental illness, a resource the likes of which are scarcely available elsewhere.  Structural imaging data are available at two timepoints approximately 2 years apart, while clinical outcome data is available across a maximum of 6 years follow-up enabling the stratification of participants on the basis of clinical outcomes (including outcomes which develop beyond the timepoint of the second MRI scan). Overall this is an interesting and unique study which is well written and of interest to the field.

 I was slightly disappointed by the application of cross-sectional image processing for this longitudinally acquired sample. Some of the “considerable heterogeneity in the direction, size and emergence of the individual brain-PADs” (raised by the authors in discussion) across the two timepoints could potentially be ameliorated by longitudinal processing, which I hope will be applied to this dataset in future. I note that the linear mixed models employed here did not take into account the time interval between scans, which while acquired ~2 years apart on average, there is variability in this timing between scans (the range was 1.0-3.8 years, mean±SD=2.12±0.35 according to the supplied supplementary datafiles) which together with the lack of inclusion of baseline age in the main exploratory model and non-linear maturation trajectories not being assessed, may impact the interpretation of the findings. Not withstanding, the analysis reported here using these simpler statistical models are valid and provide interesting new data worthy of publication, as long as the limitations are clear.  Incorporation of more complex statistical models which allow the simultaneous modelling of age, age ^2^, time (between scans), and their interactions with diagnostic group – plus incorporation of time of diagnosis (in relation to date of scan acquisition) – will allow for greater resolution of the biological processes underpinning cortical trajectory differences in future analyses. This may be particularly important given the relationship between age at baseline and brain-PAD observed here, which suggests that the differences in cortical trajectories are more apparent at earlier time points.

 The discussion describes and appropriately acknowledges the limitations of the study, particularly in regards to the low correlation with chronological age, the potential of medication effects (not examined but small exposed group), the potential for increased error in the HR-MD group due to exclusion of those individuals in the training set, and model transferability, the low resolution 1.5T scanner.

 In regards to the aging model, there are a few limitations of which to be particularly cognisant. The aging model developed employed training on scans which were also used as targets for prediction, though I note that leave-one-out prediction was employed (for HR-well and C-well scans which were employed as training data) to ameliorate circularity of the model prediction. Of course, in a perfect world, it would be much better to train the model using an independent dataset to ensure the training and test sets are unique and to reduce potential for bias. However employing a training dataset acquired on a different scanner/s may also introduce bias and reduce model fit, so there are advantages to the approach taken by the authors in this regard, particularly if no alternative dataset with equivalent acquisition parameters was available for training. The authors' discussion of possible contributors to the low correlation reported here between chronological age and brain-age raised important and relevant points, and I agree that the tight age window (15-30 years) of the training dataset may result in a poorer correlation coefficient than models trained on samples with greater age variability due to subtler age-related brain differences over a smaller developmental window. It would be interesting to see whether the findings reported here are robust with the application of different (independent) brain-age estimators, such as that developed by the ENIGMA MDD working group (Han et al, Mol Psychiatry. 2020 ^1^), although I do not suggest that as necessary for the publication of the current work. Furthermore, I note that the training set for the current study did not stratify based on gender, which may also contribute to poorer model fit due to sex-differences in brain maturation (nor did it appear to exclude relatives which may introduce artifact due to heritability of brain structures), and may warrant a further note in the discussion.

 Can the authors please clarify: 1) whether relatives were included in the training dataset for model predictor; 2) at what stage the brain-PAD values were transformed to z-scores (i.e. was this done for all values generated for scan 1 and scan 2 simultaneously, or separately for scan 1 and then scan 2 data?); 3) the relationship between CAT12 quality assurance value and accuracy of predicted brain-PAD, particularly in regards to lower bounds for ‘satisfactory’, ‘good’ and ‘excellent’ are 70%, 80%, and 90% respectively.

 Minor comment: there are still a few instances of “ageing” rather than “aging” remaining in the document (abstract background, intro para 4 (twice), intro para 8, & Fig 2 title).

Is the work clearly and accurately presented and does it cite the current literature?

Yes

If applicable, is the statistical analysis and its interpretation appropriate?

Partly

Are all the source data underlying the results available to ensure full reproducibility?

Partly

Is the study design appropriate and is the work technically sound?

Yes

Are the conclusions drawn adequately supported by the results?

Partly

Are sufficient details of methods and analysis provided to allow replication by others?

Yes

Reviewer Expertise:

Young people at increased genetic risk of bipolar disorder

I confirm that I have read this submission and believe that I have an appropriate level of expertise to confirm that it is of an acceptable scientific standard, however I have significant reservations, as outlined above.

## References

[1] : Brain aging in major depressive disorder: results from the ENIGMA major depressive disorder working group. *Mol Psychiatry*.2020; 10.1038/s41380-020-0754-0 10.1038/s41380-020-0754-0 32424236PMC8589647

# Response

**Published:** 2020-10-25

**Authors:** Laura de Nooij

We thank the reviewer for the time and effort to evaluate our manuscript and for her valuable comments and suggestions.

 Regarding the statistical analysis we acknowledge the additional limitations raised by the reviewer, including cross-sectional image processing, linear age predictors and variation in scanning interval. Future studies should apply more complex and more detailed statistical models, preferably on larger datasets in order to prevent overfitting and decrease the impact of data loss when applying longitudinal processing.

 We appreciate that the reviewer acknowledges not only the limitations but also the advantages of our leave-one-out approach. We would like to add that we had also tested a different, externally validated brainage model (brainageR version 1, https://github.com/james-cole/brainageR) for this sample. This model was developed using a large adult sample (n=2001, age mean age = 36.95 ± 18.12, age range 18–90 years). This model however appeared more biased than our previous model and showed structural overestimations for our specific sample, and therefore less optimal for our purposes. To be specific, performance metrics with brainageR (as assessed within our model’s training sample) were r = 0.39 for the correlation between brain age prediction and chronological age; MAE = 8.2 years; M brain age prediction = 30.1 (with sample M age = 22.4), SD brain age prediction = 6.3 years (with sample SD age = 3.0 years). This supports our notion that the demographics of our dataset complicated the use of externally validated models, hence our current approach.  

 The reviewer furthermore requested clarification on three matters, which we provide below:

 1) Were relatives included in the training dataset for model predictor? In the model training phase we only left out scans of the subject for whom the brain age was being predicted. So, in some cases the scans of a sibling remained in the training set. We acknowledge this limitation but would also like to note that relatedness within the sample is limited and that we did take it into account within subsequent statistical models (i.e. as random effect).

 2) At what stage the brain-PAD values were transformed to z-scores? This was done when running the statistical models (i.e. R formula: lme(scale(BrainPAD) ~ Wave * Group, random = ~ 1|PedID/ID, data=…). Given the long data format, scaling was done over all observations (i.e. across timepoints/waves). This is consistent with the approach of a single brain age prediction model (balanced with regard to timepoints). Furthermore, when one would scale separately per timepoint, the different scaling factor per timepoint would complicate interpretation.

 3) What is the relationship between CAT12 quality assurance value and accuracy of predicted brain-PAD, particularly in regards to lower bounds for ‘satisfactory’, ‘good’ and ‘excellent’ are 70%, 80%, and 90% respectively? This is an interesting question to raise. We have now explored the relationship between Brain-PAD and image quality. We have created plots, which can be viewed in the OSF repository ( https://osf.io/pz2x5/) under ‘Supplementary information’ > ‘Plots_peer_review’. The plots and corresponding statistics show no correlation between image quality and Brain-PAD (T1: r = 0.056, *p* = 0.42; T2: r = -0.086, *p *= 0.32). To elaborate further, visual inspection suggest a minor, non-statistically significant trend in which higher image quality is associated with slightly more accurate brain age predictions / less bias, because (i) at the right end the regression lines approach brain-PAD = 0, and (ii) the SD of brain-PAD appears smaller when image quality is better. This logically reflects slightly (not significantly) more noise in the model with lower quality images.

 As described above, we have updated our OSF repository by adding additional plots (and code for these plots). Furthermore, following the minor comment of inconsistent spelling (ageing/aging) we have updated our manuscript accordingly.

# Peer review: Reviewer response for version 2

**DOI:** 10.21956/wellcomeopenres.17743.r39782

**Published:** 2020-08-12

**Authors:** Manpreet K. Singh

**Affiliations:** 1 Department of Psychiatry and Behavioral Sciences, Stanford University School of Medicine, Stanford, CA, USA

Thank you for addressing reviewer comments.

Is the work clearly and accurately presented and does it cite the current literature?

No

If applicable, is the statistical analysis and its interpretation appropriate?

Yes

Are all the source data underlying the results available to ensure full reproducibility?

No

Is the study design appropriate and is the work technically sound?

Yes

Are the conclusions drawn adequately supported by the results?

Yes

Are sufficient details of methods and analysis provided to allow replication by others?

No

Reviewer Expertise:

Imaging in youth at risk for mood disorders

I confirm that I have read this submission and believe that I have an appropriate level of expertise to confirm that it is of an acceptable scientific standard.

# Peer review: Reviewer response for version 1

**DOI:** 10.21956/wellcomeopenres.17108.r38282

**Published:** 2020-05-27

**Authors:** Manpreet K. Singh

**Affiliations:** 1 Department of Psychiatry and Behavioral Sciences, Stanford University School of Medicine, Stanford, CA, USA

Summary: This is an interesting and well-written article examining structural trajectories in brain age in young individuals at familial risk for mood disorder. That the study was insufficiently sampled to assess the contribution of family history as an independent factor is a significant limitation to this study. Given that the sample involves young people with a family history of bipolar disorder, consider that as a more accurate representation of the title.

 Methods: Please specify more clearly in the methods that the time to follow up is 2 years. The design of the study is confusing and circuitous that it doesn’t become apparent until the results that the time elapsed between baseline and follow up scans is 2 years. The difference between a 15 year old two years later is significantly different than a 30 year old 2 years later in terms of brain maturation. By which standard is 15-30 years of age considered to be a “tight” age range? 

 Methods: Please clarify the temporal reference for group classifications – is it at 2 year follow up or at 6 year follow up?  Please provide justification for approach.

 Methods: Was mania severity assessed in this sample on clinical follow up?

 Results/Discussion: The HAMD scores upon follow up in the individuals categorized as HR-MD are quite low and in the upper limit of the mild range, with a mean that doesn’t even reach the lower bound of the mild range. How do authors conceptualize these individuals relative to the healthy individuals in terms of clinical staging in the context of brain age? Is deceleration of brain age likely to be associated with an increased risk for clinical progression or precede early clinical progression?  What kind of study design would be able to tease this apart? It would be helpful to include rational future directions beyond increasing sample size.

 Scanning at 1.5 tesla significantly limits spatial resolution and should be noted as a limitation. 

 Minor comment: unless specified by the journal style, the term “ageing” spelled in this way is distracting.

Is the work clearly and accurately presented and does it cite the current literature?

No

If applicable, is the statistical analysis and its interpretation appropriate?

Yes

Are all the source data underlying the results available to ensure full reproducibility?

No

Is the study design appropriate and is the work technically sound?

Yes

Are the conclusions drawn adequately supported by the results?

Yes

Are sufficient details of methods and analysis provided to allow replication by others?

No

Reviewer Expertise:

Imaging in youth at risk for mood disorders

I confirm that I have read this submission and believe that I have an appropriate level of expertise to confirm that it is of an acceptable scientific standard, however I have significant reservations, as outlined above.

# Response

**Published:** 2020-07-14

**Authors:** Laura de Nooij

We thank the reviewer for her time and effort in reviewing our article and her constructive comments. Based on these comments, we have improved the manuscript and have uploaded a revised version of this article. Here, we would also like to address the comments on a point-by-point basis.

*"This is an interesting and well-written article examining structural trajectories in brain age in young individuals at familial risk for mood disorder. That the study was insufficiently sampled to assess the contribution of family history as an independent factor is a significant limitation to this study."*

As above, we are happy that these particular limitations of the study are clearly portrayed and our rationale transparent. In particular, we provide detailed explanations for the choice of within-sample prediction, our concerns about just using the control sample alone, further, we thoroughly tested performance of the model and overfitting per group, we tested other available algorithms, and tested the method against other widely available brain age prediction software derived in independent samples (BrainAge). We therefore consider that though the study has limitations, and we acknowledge the need for replication, it represents an important foundation for further research in the field.

*"Given that the sample involves young people with a family history of bipolar disorder, consider that as a more accurate representation of the title"*

  We agree with the reviewer and have added the cohort name to the title of the manuscript for clarity, this now reads “Longitudinal trajectories of brain age in young individuals at familial risk of mood disorder from the Scottish Bipolar Family Study”

*"Methods: Please specify more clearly in the methods that the time to follow up is 2 years. The design of the study is confusing and circuitous that it doesn’t become apparent until the results that the time elapsed between baseline and follow up scans is 2 years. The difference between a 15 year old two years later is significantly different than a 30 year old 2 years later in terms of brain maturation. By which standard is 15-30 years of age considered to be a “tight” age range?"*

We have now clarified in the methods that the time between scans was ~ 2 years.  We also agree with the reviewer that the change over time for a 15 year old will be different than that for someone aged 30 years. For this reason, we performed an age interaction model, investigating the potential effect of age on these relationships. These results were initially included as exploratory findings in supplemental information and indeed demonstrated larger decelerations for younger participants in the HR-MD group. These results are now detailed in the main body of the manuscript.

*"Methods: Please clarify the temporal reference for group classifications – is it at 2 year follow up or at 6 year follow up?  Please provide justification for approach."*

This information has now been added to the manuscript. In most cases individuals were seen every 2 years for a total of 6 years (x3 assessments, but only 2 scans)

For the control group, individuals were included if they had remained without being diagnosed with any psychiatric disorder throughout the period of the study (via assessments and/or GP records). Those that did become unwell were a small group (n=12) and are not included in the current analysis. This approach was used to reduce heterogeneity in the control group.

For the high-risk group, none met clinical criteria for any mood disorder at baseline. If at any assessment they did meet criteria for a mood disorder (at the time of assessment or over the intervening period since the earlier assessment) they were considered as being in the HR-MD group. So, once someone had a diagnosis, even though they may not have been actively symptomatic at the time of the assessment, they were still considered as being in the mood disorder group. This approach was based on the premise that once an individual had met diagnostic criteria at any time over the course of the study, they were no longer ‘high risk’ for mood disorder, but actually a ‘case’.

*"Methods: Was mania severity assessed in this sample on clinical follow up?"*

Yes, mania was also assessed at every assessment using the Young Mania Rating Scale (YMRS), however previous studies indicated that there were no significant differences between the three groups in terms of the YMRS cross sectionally or over time, and the median and interquartile ranges were low, hence we did not specifically examine mania ratings in the current analysis (Papmeyer et al, Psychiatry Res. 2016 Feb 28; 248: 119–125; Papmeyer et al, Psychol Med. 2015 Nov; 45(15): 3317–3327.). This information has now been added to the manuscript.

*"Results/Discussion: The HAMD scores upon follow up in the individuals categorized as HR-MD are quite low and in the upper limit of the mild range, with a mean that doesn’t even reach the lower bound of the mild range. How do authors conceptualize these individuals relative to the healthy individuals in terms of clinical staging in the context of brain age? Is deceleration of brain age likely to be associated with an increased risk for clinical progression or precede early clinical progression?  What kind of study design would be able to tease this apart? It would be helpful to include rational future directions beyond increasing sample size."*

The reviewer is correct that the HAMD scores are relatively low, we however note that the depression status was not defined according to the severity of current symptoms at the time of the scan, but based on lifetime depression, a note to this effect has been added to the discussion.

Regarding the second point, it is not possible to disentangle whether the differences in brain age are a cause or consequence of clinical progression. In future studies, earlier scans prior to prodromal phases of disease, along with leveraging opportunities from other statistical approaches to determine causal directionality could be implemented to attempt to understand these relationships. This has also now been added to the discussion.

*"Scanning at 1.5 tesla significantly limits spatial resolution and should be noted as a limitation."*

This is now explicitly mentioned as a limitation in the manuscript. As above we also now acknowledge that though this is not ideal given current technology, the choice of scanner strength was determined at the beginning of this 10 year prospective study. We considered the value of having prospective longitudinal data, together with detailed QC (using software quality assurance metrics in combination with manual checks excluding scans with artefacts or of otherwise insufficient quality) provided sufficient confidence in the data quality used in the current study.

*"Minor comment: unless specified by the journal style, the term “ageing” spelled in this way is distracting."*

All spellings of the term “ageing” have now been changed to “aging”

# Peer review: Reviewer response for version 1

**DOI:** 10.21956/wellcomeopenres.17108.r37434

**Published:** 2020-01-20

**Authors:** Philip B. Mitchell

**Affiliations:** 1 School of Psychiatry, University of New South Wales, Sydney, NSW, Australia

This is the first study of cross-sectional and longitudinal changes in brain aging in young people at high genetic risk of bipolar disorder. As such, it is of interest to the field.

 The main result is the somewhat counter-intuitive finding that those at risk who go on to develop a mood disorder demonstrate reduced ('decelerated') brain aging. This contrasts with findings in older patients in which depression is related to increased brain aging. The explanation for this unexpected finding is less than convincing.

 The authors are frank about the limitations of this study, the major one being that the 'training' dataset was a combination of controls who stayed well, and at risk subjects who stayed well - thereby confounding risk and clinical status.

 Issues to be addressed:

1. There needs to be more frank acknowledgement that the brain aging difference in the risk group that developed mood episodes may have been a result of the depression in addition to the risk state per se.
2. The study used a 1.5T MRI scanner. Is it possible that this did not provide sufficient resolution for such a study?

Is the work clearly and accurately presented and does it cite the current literature?

Yes

If applicable, is the statistical analysis and its interpretation appropriate?

Yes

Are all the source data underlying the results available to ensure full reproducibility?

No source data required

Is the study design appropriate and is the work technically sound?

Yes

Are the conclusions drawn adequately supported by the results?

Partly

Are sufficient details of methods and analysis provided to allow replication by others?

Yes

Reviewer Expertise:

Young people at increased genetic risk of bipolar disorder

I confirm that I have read this submission and believe that I have an appropriate level of expertise to confirm that it is of an acceptable scientific standard, however I have significant reservations, as outlined above.

# Response

**Published:** 2020-07-14

**Authors:** Laura de Nooij

We thank the reviewer for his time and effort in reviewing our article and his supportive and positive comments. Based on these comments, we have improved the manuscript and have uploaded a revised version of this article. Here, we would also like to address the comments on a point-by-point basis.

*"The main result is the somewhat counter-intuitive finding that those at risk who go on to develop a mood disorder demonstrate reduced ('decelerated') brain aging. This contrasts with findings in older patients in which depression is related to increased brain aging. The explanation for this unexpected finding is less than convincing."*

 We have now expanded on this finding and simplified the discussion, adding that the majority of studies reporting accelerated brain aging in mood disorder are conducted in older adult samples, rather than younger adolescent cohorts. Given the non-linear trajectory of regional brain maturation / aging over the life-course, particularly over periods where there is significant developmental change (e.g. adolescence), it may not be the case that there is a simple retrospective linear trajectory of accelerated biological aging from these adult studies to periods earlier in life. Further, this finding fits with wider theories of adolescent psychopathology, for example the ‘dual system’ model where delayed maturation (‘decelerated brain age’) in higher order cortical regions in relation to limbic regions is proposed to underlie difficulties in emotional regulation, cognitive function and social behaviour, increasing vulnerability to mood disorder.

*"The authors are frank about the limitations of this study, the major one being that the 'training' dataset was a combination of controls who stayed well, and at risk subjects who stayed well - thereby confounding risk and clinical status."*

 We are happy that these particular limitations of the study are clearly portrayed and our rationale transparent. In particular, we provide detailed explanations for the choice of within-sample prediction, our concerns about just using the control sample alone, further, we thoroughly tested performance of the model and overfitting per group, we tested other available algorithms, and tested the method against other widely available brain age prediction software derived in independent samples (BrainAge). We therefore consider that though the study has limitations, and we acknowledge the need for replication, it represents an important foundation for further research in the field.

*"There needs to be more frank acknowledgement that the brain aging difference in the risk group that developed mood episodes may have been a result of the depression in addition to the risk state per se."*

 We have now added a section to the discussion addressing this point, explaining that the brain aging differences may have resulted from depression and risk state in combination, and that we cannot disentangle effects of risk and depressive symptoms in the current study.

*"The study used a 1.5T MRI scanner. Is it possible that this did not provide sufficient resolution for such a study?"*

 We also now acknowledge this point in the limitations section. Though this is not ideal given current technology, the choice of scanner strength was determined at the beginning of this 10 year prospective study. We considered the value of having prospective longitudinal data, together with detailed QC (using software quality assurance metrics in combination with manual checks excluding scans with artefacts or of otherwise insufficient quality) provided sufficient confidence in the data quality used in the current study **.**

 To elaborate from a technical point of view, 1.5T MRI scans indeed provide more noisy data and lower resolution than 3T MRI data. Smoothing is a recommended pre-processing step that reduces the noise on voxel level and was also applied in the pipeline of the current study. Subsequently, in order to appropriately deal with this high-dimensional data, voxels were resampled into greater voxel sizes in order to reduce the number of variables (features) for the machine learning approach (note however that due to previous smoothing, this step did not further reduce the resolution). We find that these recommended pre-processing steps reduce noise and resolution, but that the resolution of our pattern recognition approach is still higher than with standard sMRI analysis approaches in which voxels are often averaged across areas. The last step of the pipeline was the extraction of features with PCA. We thus applied multiple pre-processing steps, and although derived and pre-processed variables will also be more precise when input variables are more precise (e.g. due to higher scanner strength), we would argue that the low scanner strength in itself does not comprise the methods of this study. In addition, we reference several other studies that applied similar approaches also including 1.5T sMRI data (e.g. Franke et al., NeuroImage, 2012, 63, 1305–1312; Gaser et al, PLOS ONE, 2013, 8(6), e67346).
